# Reprogrammable Metamaterial Processors for Soft Machines

**DOI:** 10.1002/advs.202305501

**Published:** 2023-12-31

**Authors:** Zhongdong Jiao, Zhenhan Hu, Zeyu Dong, Wei Tang, Huayong Yang, Jun Zou

**Affiliations:** ^1^ State Key Laboratory of Fluid Power and Mechatronic Systems Zhejiang University Hangzhou 310058 China

**Keywords:** metamaterials, reprogrammable, soft machines, soft materials, soft robots

## Abstract

Soft metamaterials have attracted extensive attention due to their remarkable properties. These materials hold the potential to program and control the morphing behavior of soft machines, however, their combination is limited by the poor reprogrammability of metamaterials and incompatible communication between them. Here, printable and recyclable soft metamaterials possessing reprogrammable embedded intelligence to regulate the morphing of soft machines are introduced. These metamaterials are constructed from interconnected and periodically arranged logic unit cells that are able to perform compound logic operations coupling multiplication and negation. The scalable computation capacity of the unit cell empowers it to simultaneously process multiple fluidic signals with different types and magnitudes, thereby allowing the execution of sophisticated and high‐level control operations. By establishing the laws of physical Boolean algebra and formulating a universal design route, soft metamaterials capable of diverse logic operations can be readily created and reprogrammed. Besides, the metamaterials' potential of directly serving as fluidic processors for soft machines is validated by constructing a soft latched demultiplexer, soft controllers capable of universal and customizable morphing programming, and a reprogrammable processor without reconnection. This work provides a facile way to create reprogrammable soft fluidic control systems to meet on‐demand requirements in dynamic situations.

## Graphical abstract

This work develops printable and recyclable soft metamaterials possessing reprogrammable embedded intelligence. These metamaterials can function as soft processors, enabling universal and customizable morphing programming as well as autonomous reprogramming for soft machines.

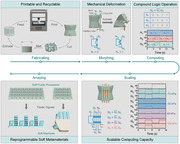

## 1 Introduction

Metamaterials, characterized by their periodic arrangement of building blocks, have emerged as a cutting‐edge frontier in the realms of science and engineering.^[^ ^1^, ^2^, ^3^, ^4^ ^]^ These materials offer programmable and exceptional properties derived from their geometric configurations rather than their intrinsic compositions. By elaborately designing the arrangement and interactions of these constituent elements, the properties of soft metamaterials, including optical,^[^ ^5^, ^6^ ^]^ acoustic,^[^ ^7^, ^8^, ^9^ ^]^ thermal,^[^ ^10^, ^11^, ^12^ ^]^ and mechanical properties,^[^ ^13^, ^14^, ^15^, ^16^ ^]^ can be continuously adjusted in a wide range. This inherent versatility enables their integration into diverse research domains such as robotics,^[^ ^17^, ^18^, ^19^ ^]^ flexible electronics,^[^ ^20^, ^21^, ^22^ ^]^ microfluidics,^[^ ^23^, ^24^, ^25^ ^]^ and chemistry.^[^ ^26^, ^27^ ^]^

Traditional pneumatic soft robots typically require microcontrollers, relays, and solenoid valves to enable actuators to achieve desired deformations.^[^ ^28^, ^29^, ^30^, ^31^, ^32^, ^33^ ^]^ However, the incorporation of these rigid components compromises the inherent compliance of soft robots. Therefore, creating fully soft, reprogrammable robots has been a grand challenge.^[^ ^34^ ^]^ In recent advancements, the combination of metamaterials and soft robotics has unlocked new avenues for imbuing robots with embedded physical intelligence. By employing metamaterials as the structure basis of robot bodies, morphing information can be coded into soft robots, enabling them to transform simple inputs into complex morphing outputs with specific sequences.^[^ ^35^, ^36^, ^37^, ^38^, ^39^ ^]^ However, these actuation sequences are typically pre‐programmed and can hardly be altered during in‐life service, limiting their applications in dynamic scenarios.

To address this challenge, a potential solution is to endow metamaterials with reprogrammable logical computation abilities, enabling them to perform various combinatorial and sequential logic operations.^[^ ^40^, ^41^, ^42^, ^43^, ^44^, ^45^, ^46^, ^47^ ^]^ However, the execution of these mechanical logic computations depends on specific inputs, such as sliding switches,^[^ ^40^ ^]^ applying compressive forces,^[^ ^41^, ^42^, ^43^, ^46^ ^]^ or exposing to specific solvents,^[^ ^44^ ^]^ which are difficult to generate autonomously. Moreover, the logic outputs of these computations cannot directly act as the actuation stimuli for soft robots. Consequently, the incompatible input and output signals pose significant challenges in regulating the movement of soft robots with existing reprogrammable metamaterials.

In this work, we present a novel class of intelligent soft metamaterials with intrinsic reprogrammability, printability, and recyclability. These metamaterials consist of interconnected and periodically arranged logic unit cells. Each unit cell is able to execute compound logic operations encompassing multiplication and negation and exhibit scalable computing capacity. By introducing the laws of physical Boolean algebra and a universal design route, these unit cells can be configured into soft metamaterials with various functions, including fundamental logic and advanced arithmetic operations. To demonstrate their potential in soft robotics, we construct a soft latched demultiplexer capable of managing 2^n^−1 independent actuators using only n fluidic inputs. Furthermore, we develop metamaterials‐based soft controllers that achieve autonomous regulation over both positive and negative pressures, customizable morphing on soft robots, and reprogrammable physical intelligence without reconnection.

## 2 Results

### 2.1 Design and Operational Principle

**Figure 1:**
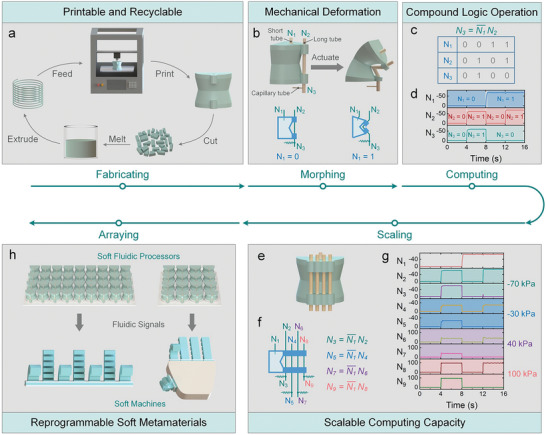
Design and operational principle of the logic unit cell. a) The logic unit cells can be printed and recycled. b) The mechanical deformation of the logic unit cell. The bottom figures are the fluidic network of the unit cell. Logic state “0” represents atmospheric pressure, while logic state “1” represents vacuum pressure. c) The truth table of the compound logic operation embedded in the unit cell. d) The pressure responses of the logic unit cell. e–g) The structure (e), fluidic network (f), and pressure responses (g) of the unit cell with scaled computation capacity. h) The logic unit cells are tessellated into soft metamaterials, which are able to function as fluidic processors for the regulation of soft machines.

We construct a logic unit cell by combining a soft airtight chamber with two elastomer tubes and a capillary tube, as shown in **Figure** **1**b. The chamber is a hollow half‐cylinder with a triangular groove in the middle (Figure S1, Supporting Information), facilitating its direct fabrication using fused deposition modeling (FDM) 3D printing technology. As demonstrated in Figure 1a, the unit cell can be printed in a commercial 3D printer with thermoplastic polyurethane (TPU) filament. TPU is a kind of melt‐processable thermoplastic elastomer and can be melted through heating, allowing the recycling of the logic unit cell. As depicted in Figure 1a and Movie S2 (Supporting Information), the process begins by fragmenting an old unit cell into small pieces. These fragments are then fed into a plastic extrusion machine. The machine melts these plastic fragments and converts them into continuous filament wires, which can be directly used to print a new logic unit cell.

As illustrated in Figure 1b, the three tubes in the unit cell form a fluidic network, in which port *N*_1_ regulates the internal pressure of the chamber, ports *N*_2_ and *N*_3_ are linked via a long elastomer tube, and the capillary tube is connected to port *N*_3_. As displayed in Figure S1c (Supporting Information), the logic unit cell is an uneven structure with varying thicknesses, where the curved surface is thinner than the flat surface. The nonuniform thickness allows the curved surface to collapse along the triangular groove under vacuum pressure, inducing bending morphing of the unit cell (Movie S1, Supporting Information). When the actuation pressure is lower than the threshold pressure *P_kink_* (Figure S3, Supporting Information), this buckling behavior causes the long elastomer tube to be kinked, thus separating ports *N*_2_ and *N*_3_ from each other.

Our logic unit cell depends on the mechanical behavior coupled with a fluidic network to execute logic operations. In Figure 1b, we present the unactuated state of the logic unit cell, wherein ports *N*_2_ and *N*_3_ are interconnected. Upon applying vacuum pressure to port *N*_1_, the connection between ports *N*_2_ and *N*_3_ is disrupted, thereby causing the pressure at port *N*_3_ to equilibrate with atmospheric pressure via the capillary tube. If vacuum pressure is denoted as logic “1”, and atmospheric pressure is denoted as logic “0”, this unit cell effectively carries out a compound logic operation that contains negation and multiplication operations, that is $N_{3}=\bar{N_{1}}N_{2}$, as shown in Figure 1c,d and Movie S1 (Supporting Information). The behavior of this fluidic network can be analyzed using an analogous electrical circuit, where the logic operation can be modeled as the charging or discharging of corresponding components within a resistor–capacitor circuit (Figure S4a–c, Supporting Information; see the “Analytical model of the logic unit cell” section in Experimental Section for details). The response time calculated with this model agrees well with the experimental values (Figure S4d,e, Supporting Information).

To investigate the frequency response of our logic unit cell, we operated it at frequencies ranging from 0.2 to 3 Hz with a vacuum pressure of −70 kPa. As shown in Figure S5 (Supporting Information), the results revealed that the highest frequency that allowed for logic computation was 2 Hz. The monolithic chamber of the unit cell also endows it with exceptional durability. In Figure S6 (Supporting Information), we conducted 10 000 cycles of operation at a frequency of 0.5 Hz, and the computation capability of the logic unit cell exhibited little variation.

The computation capacity of a single unit cell can be scaled up by augmenting the number of long elastomer tubes along the curved surface. As illustrated in Figure 1e,f, a logic unit cell equipped with four long tubes is able to execute four logic operations simultaneously. This scalable capability also makes it possible to regulate multiple fluidic pressures with different pressure types and magnitudes. In Figure 1g and Movie S3 (Supporting Information), we demonstrate this functionality by effectively processing four pressures (−70, −30, 40, and 100 kPa) using a single unit cell.

Both the input and output signals of the logic unit cell are fluidic pressures, therefore tessellating these logic unit cells into an array and elaborately connecting them through fluidic channels will yield a reprogrammable and intelligent soft metamaterial with built‐in fluidic network. This interconnected soft metamaterial is able to produce fluidic signals directly applicable to the regulation of soft machines, just like fluidic processors, as illustrated in Figure 1h. Besides, the reconfigurable fluidic networks allow the generated fluidic signals to be reprogrammed in an on‐demand manner.

### 2.2 Laws of Physical Boolean Algebra

A single unit cell is capable of exhibiting a compound logic operation coupling negation and multiplication. However, it is still challenging to perform other logic operations with this compound logic operation unit cell. Here, we propose the laws of physical Boolean algebra, which enable the conversion of any logic expressions into physical logic unit cells. According to the laws of Boolean algebra, any logic expression can be expressed as a combination of negation, multiplication, and addition operations. Consequently, we will demonstrate how to execute the three types of fundamental logic operations with our logic unit cells in the following section.

**Figure 2:**
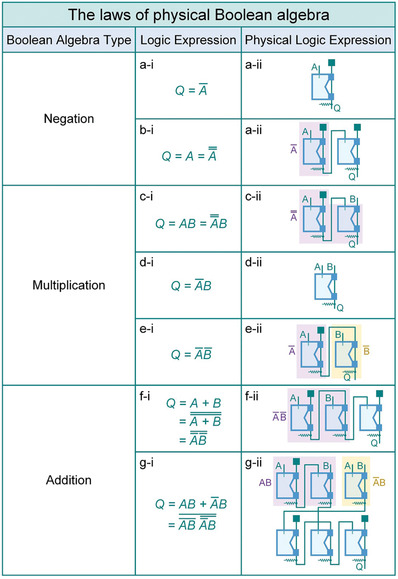
The laws of physical Boolean algebra. a) The Boolean expression (a)‐i and fluidic network (a)‐ii of the single negation operation. b) The double negation operation. c) The multiplication operation of two normal logic variables. d) The multiplication operation of a normal logic variable and an inverted logic variable. e) The multiplication operation of two inverted logic variables. f) The addition operation of two logic variables. g) The addition operation of two logic expressions. The dark cyan block represents vacuum pressure.

As demonstrated in **Figure** **2**a, the negation operation can be realized by connecting port *N*_2_ to a vacuum pressure, with ports *N*_1_ and *N*_3_ serving as the input and output variables, respectively. The double negation operation can be accomplished by connecting two unit cells in series, where the port *N*_3_ of the first unit cell is connected to the port *N_1_* of the second unit cell (Figure 2b).

The multiplication can be enabled by combining the inherent compound logic operation with the negation operation. For example, a multiplication operation involving two normal logic variables can be converted into an expression in which the first logic variable is double inverted (Figure 2c). Then the port *N*_1_ of the first unit cell and the port *N*_2_ of the second unit cell are taken as the two input variables. If one inverted logic variable is multiplied by one normal logic variable, the required logic gates can be reduced to one by setting port *N*_1_ as the inverted logic variable and port *N*_2_ as the normal logic variable (Figure 2d). When two inverted logic variables are multiplied, the port *N*_3_ of the first unit cell is connected to the port *N*_2_ of the second unit cell (Figure 2e).

As for the addition operation, the expression is supposed to be converted into negation and multiplication operations according to De Morgan's theorem, as illustrated in Figure 2f,g. Although the addition operation can also be achieved by directly connecting these terms in parallel, this connection may lead to incorrect results in some cases. For example, the logic expression $Q=\bar{A}B+\bar{C}D$ can be physicalized in parallel form, as demonstrated in Figure S7 (Supporting Information). When ports *B* and *D* are connected to the vacuum pressure, the generated results are correct. If input ports *A*, *B*, *C* are connected to atmospheric pressure and input port *D* is connected to the vacuum pressure, the output port *Q* is supposed to be in the logic “1” state. However, the parallel connection interconnects ports *B* and *D*, resulting in the vacuum source being connected to the atmosphere. Consequently, output port *Q* becomes a logic “0” state, which is a false output.

### 2.3 Fundamental Logic Gates

**Figure 3:**
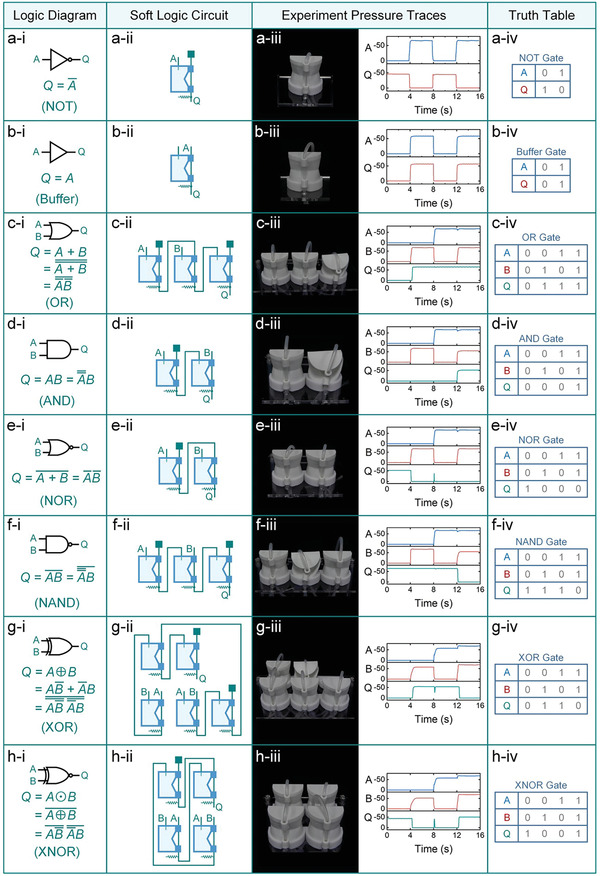
The fundamental logic gates are enabled by the soft logic unit cells. (a, i) The logic symbol and Boolean expression of the NOT gate. (a, ii) The fluidic network of the NOT gate. (a, iii) The experimental image and pressure traces of the NOT gate. (a, iv) The truth table of the NOT gate. b) The Buffer gate. c) The OR gate. d) The AND gate. e) The NOR gate. f) The NAND gate. g) The XOR gate. h) The XNOR gate.

Using the laws of physical Boolean algebra, we are able to build a complete set of fundamental logic gates, including 1‐bit gates such as NOT and Buffer, as well as 2‐bit gates: OR, AND, NOR, NAND, XOR, and XNOR (Movie S4, Supporting Information). As shown in **Figure** **3**a, the NOT gate can be created with a single unit cell, where input *A* is connected to port *N*_1_, the vacuum source is connected to port *N*_2_, and output *Q* is connected to port *N*_3_. The Buffer gate can be expressed by connecting ports *N*_1_, *N*_2_, and *N*_3_ to atmospheric pressure, input *A*, and output *Q*, respectively (Figure 3b).

The OR gate, an addition operation, can be constructed by converting it into one multiplication and three negation operations (Figure 3c). The AND gate, the multiplication of two variables, can be physicalized by double negating one of the variables (Figure 3d). The NOR and NAND gates are the inverted OR and AND gates, respectively. Therefore, they can be physically implemented by removing (Figure 3e) or adding (Figure 3f) a negation operation from OR and AND gates. The XOR gate contains addition, multiplication, and negation operations. By converting addition operations into multiplication and negation operations, this gate can be physicalized (Figure 3g). Similarly, the XNOR gate is an inverted XOR gate and can also be constructed by removing a negation operation from the XOR gate, as demonstrated in Figure 3h.

### 2.4 Universal Design Route

**Figure 4:**
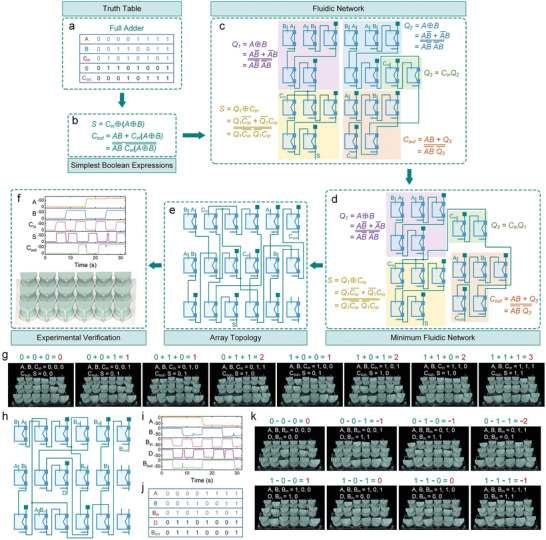
The universal design route of soft fluidic metamaterials. a) The truth table of the full adder. b) The simplest Boolean expression of the full adder. c) The fluidic network of the full adder. d) The minimum fluidic network of the full adder. e) The fluidic network is rearranged in the form of an array topology. f) The pressure traces and schematic illustration of the metamaterial‐based full adder. g) All possible configurations of the metamaterial‐based full adder. h) The array fluidic network of the full subtractor. i) The pressure traces of the metamaterial‐based full subtractor. j) The truth table of the full subtractor. k) All possible configurations of the full subtractor.

Leveraging the fundamental logic gates and the laws of physical Boolean algebra, any higher‐level combinational logic circuit can be constructed utilizing the logic unit cells. However, the physicalization of complex logic circuits poses challenges, as it is error‐prone and time‐consuming. A complex logic circuit can be expressed with several forms of fluidic networks, and the network with the minimum number of unit cells is preferred. To achieve this, we formulate a universal design route to obtain the simplest fluidic networks, and the soft full adder circuit is taken as an example (**Figure** **4**a–g).

The full adder performs the addition operation on 1‐bit numbers *A*, *B*, and *C*_in_ (carry input) and yields a 2‐bit number, where *S* and *C*_out_ (carry output) correspond to the first‐bit and second‐bit digits. Then the truth table of the full adder is obtained based on addition rules (Figure 4a). Using this truth table, the two outputs are expressed as the simplest Boolean functions without addition operation (Figure 4b). Next, the simplest Boolean functions are transformed into the fluidic network, as shown in Figure 4c. By merging identical terms within the two Boolean functions, the number of unit cells is minimized (Figure 4d). Subsequently, the fluidic network is arranged in an array and physicalized with logic unit cells, thereby forming a soft fluidic metamaterial (Figure 4e). Finally, all possible configurations of the full adder are validated in the physical metamaterial, as depicted in Figure 4f,g and Movie S5 (Supporting Information). Furthermore, we also built a soft metamaterial capable of executing the full subtractor operation (Figure 4h). Experimental results revealed that the fluidic logic outputs are consistent with the corresponding truth table (Figure 4i–k; Movie S6, Supporting Information), implying the universality of this design approach.

### 2.5 Metamaterial Processors

#### 2.5.1 Soft Fluidic Demultiplexer

**Figure 5:**
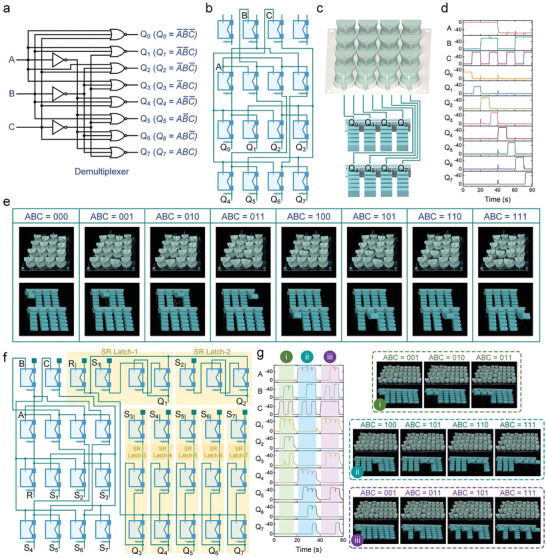
The metamaterial‐based fluidic demultiplexer. a) The logic circuit of the demultiplexer. b) The array fluidic network of the soft demultiplexer. c) The demultiplexer acts as the fluidic processor for a soft machine with eight bending actuators. d) The pressure traces of the soft fluidic demultiplexer. e) The demultiplexer governs the morphing of eight bending actuators. f) The array fluidic network of the soft latched demultiplexer. g) The latched demultiplexer is able to set seven soft actuators to any desired combination of actuated or unactuated states. In the first combination, the first, second, and third actuators are in actuated states. In the second combination, the fourth, fifth, sixth, and seventh actuators are in actuated states. In the third combination, the first, third, fifth, and seventh actuators are in actuated states.

With the successful execution of complex logic operations by the presented soft metamaterials, harnessing this innovative technology to construct fluidic processors for the control of soft robots has become a viable possibility. To demonstrate this capability, we have developed a soft fluidic demultiplexer, which allows three inputs to govern eight mutually exclusive outputs, as illustrated in **Figure** **5**a. The eight outputs can be expressed as a combination of multiplication and negation operations based on the three inputs. Then a soft metamaterial with an array of 4×4 is employed to construct the fluidic demultiplexer (Figure 5b). The eight outputs are connected to eight independent bending actuators, while the logic states of the three inputs are regulated by three solenoid valves (Figure 5c). By systematically cycling the demultiplexer through all eight addresses in binary counting order, the eight outputs are sequentially transitioned into logic “1” state (vacuum state), thereby causing the corresponding actuators to bend upward (Figure 5d,e; Movie S7, Supporting Information). In general, this approach can control 2^n^ actuators using n computer‐controlled solenoid valves. However, the inherent mutually exclusive outputs of the demultiplexer restrict it to controlling multiple actuators at the same time.

To overcome this limitation, we then devised a soft latched demultiplexer using the soft metamaterials. As depicted in Figure 5f, the outputs of the demultiplexer are routed to 7 SR latches. The first output of the demultiplexer is connected to the common “Reset” port of these SR latches, while the remaining 7 outputs are linked to the “Set” ports. In this way, the soft metamaterial can set soft actuators to any desired combination of actuated or unactuated states, with the actuators retaining their states until a different state is set, as shown in Figure 5g and Movie S8 (Supporting Information). This latched demultiplexer is able to regulate 2^n^−1 independent actuators using n solenoid valves, significantly reducing the hardware requirements for managing multiple actuators in fluidic soft robots.

#### 2.5.2 Universal Autonomous Soft Controller

**Figure 6:**
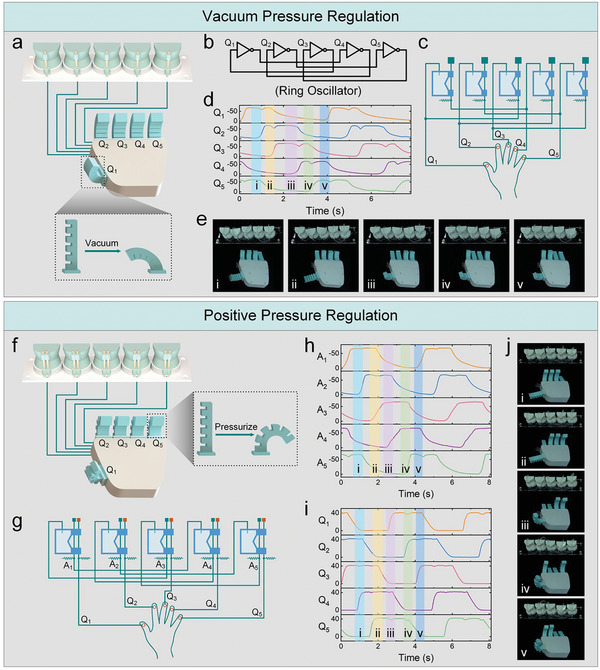
The metamaterial‐based autonomous soft controller. a) The soft ring oscillator with five unit cells regulates a vacuum‐powered soft hand. b) The logic circuit of the five‐stage ring oscillator. c) The fluidic network of the soft ring oscillator and vacuum‐powered hand. d) The pressure traces of the soft ring oscillator. e) The five fingers are evacuated and restored sequentially. f) The soft ring oscillator regulates a positive pressure‐driven soft hand. Each unit cell in this configuration has two long tubes along the curved surface. g) The fluidic network of the soft ring oscillator and positive pressure‐driven hand. h, i) The output pressure traces of the ring oscillator (h) and the internal pressure traces of the soft hand (i). j) The five fingers are pressurized and restored sequentially.

The intelligent soft metamaterials can also be used to build autonomous fluidic processors that operate independently of off‐board solenoid valves. As shown in **Figure** **6**a,c, we construct a soft five‐stage ring oscillator^[^ ^48^ ^]^ consisting of five interconnected logic unit cells. When all the *N*_2_ ports are exposed to vacuum pressure, the five‐unit cells undergo periodic collapse and restoration, leading to the generation of cyclic and oscillatory pressures at the five *N*_3_ ports. These *N*_3_ ports, designated as *Q*_1_, *Q*_2_, *Q*_3_, *Q*_4_, and *Q*_5_ in Figure 6c, are connected to the five fingers of a soft hand. The periodic oscillatory pressures enable these fingers to extend/bend sequentially (Figure 6d,e; Movie S9, Supporting Information). This soft metamaterial only requires a constant vacuum pressure to enable control over soft robots, eliminating the necessity for additional rigid valves.

Despite being powered by vacuum pressure, the scalable computation capacity of the unit cell allows the soft metamaterial to govern soft actuators driven by positive pressure. In this case, both the positive and negative pressures are denoted as logic “1” state. As shown in Figure 6f, the previous unit cells are replaced by those containing two long tubes to scale up the computation capacity of soft metamaterials. In each unit cell, one long tube is employed to construct a soft ring oscillator circuit, while the other one is responsible for providing positive pressure for soft actuators (Figure 6g; Movie S9, Supporting Information). In the unactuated state of the logic unit cell, the positive pressure induces the bending of the soft finger. Conversely, when the unit cell is actuated, both long tubes collapse simultaneously, causing the finger connecting with the kinked long tube to return to its initial state. In this way, the soft metamaterial produces five oscillatory positive pressures, which can be directly used to actuate five soft fingers to extend/bend sequentially and periodically (Figure 6h–j).

#### 2.5.3 Customizable Morphing Programming

**Figure 7:**
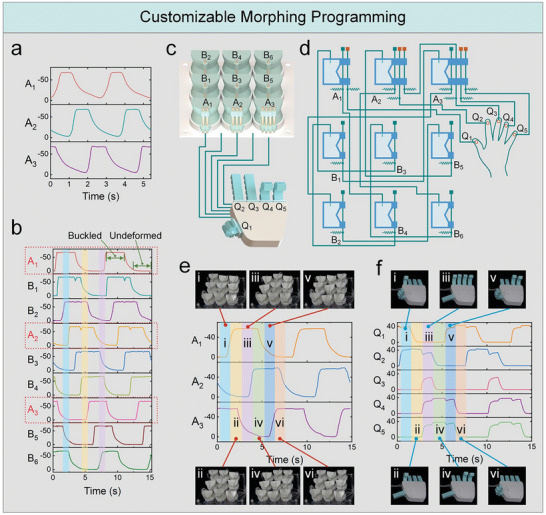
The customizable morphing programming of the soft metamaterial processor. a) The pressure responses of a soft three‐stage ring oscillator. b) The pressure responses of a soft nine‐stage ring oscillator. c) The soft metamaterial is utilized to program the gesture of a soft hand. d) The fluidic network of the soft metamaterial and hand. e) The deformation and pressure responses of the soft metamaterial while the soft hand is exhibiting different gestures. f) The soft hand exhibits six different gestures under the management of the soft metamaterial. The middle figure is the pressure responses of the soft hand. (f, i) “OK” sign; (f, ii) Index finger bent; (f, iii) Thumb‐up gesture; (f, iv) “Three” gesture; (f, v) “V for victory” sign; (f, vi) “Four” gesture.

The soft ring oscillator depends on the interconnections among logic unit cells to generate periodic and oscillatory pressures. Nevertheless, the simultaneous deformation of adjacent unit cells and adjacent fingers (Figure 6d,h,i) restricts the programming capability of the metamaterial processors. This issue can be solved by adding unit cells between adjacent unit cells, thereby decoupling their deformations, as depicted in **Figure** **7**a,b. Compared with the three‐stage ring oscillator, the nine‐stage ring oscillator possesses longer stable phases (the fully undeformed and buckled states). This allows unit cells *A*_1_, *A*_2_, and *A*_3_ to be in the buckled state individually (the colored areas in Figure 7b). Combining this decoupling strategy with the scalable computation capacity, the metamaterial processors are able to program the morphing behavior of soft machines as required.

As demonstrated in Figure 7c,d and Movie S10 (Supporting Information), a soft metamaterial with an array of 3×3 is harnessed to control a soft hand to exhibit six hand gestures. In this fluidic network, unit cells *A*_1_–*A*_3_ are responsible for regulating the morphing of the soft hand, while unit cells *B*_1_–*B*_6_ serve to prolong the stable phases of unit cells *A*_1_–*A*_3_. As illustrated in Figure 7e(iii), the decoupling strategy allows only one unit cell (*A*_1_) to be in the buckled state, resulting in the thumb‐up gesture (Figure 7f(iii)). Leveraging the scalable computation capacity, the hand can exhibit gestures with multiple fingers extended, including the “OK” sign (Figure 7f(i)), “V for victory” sign (Figure 7f(v)), “Four” gesture (Figure 7f(vi)), and so on. The ability to generate autonomous and customizable fluidic signals holds promise for achieving sophisticated and high‐level control operations in soft machine systems.

#### 2.5.4 Reprogrammable Physical Intelligence

**Figure 8:**
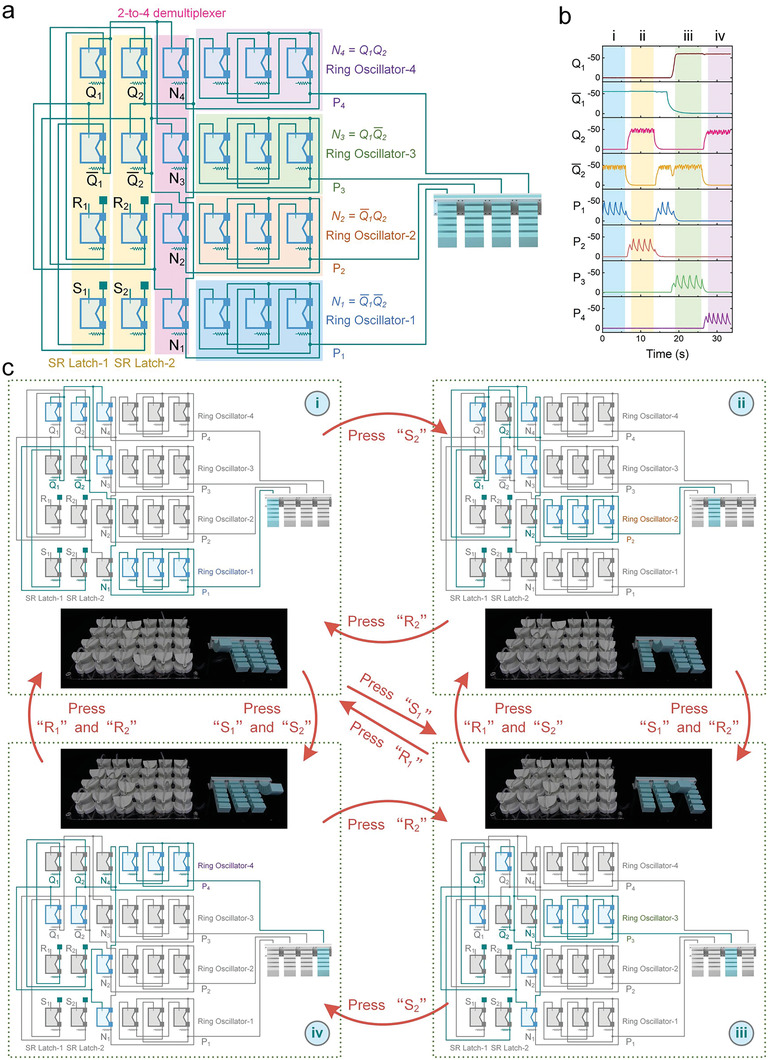
Autonomously reprogrammable soft metamaterial processor. a) The fluidic network of the soft processor and fingers. b) The pressure responses of the soft processor. c) The processor can be transitioned between four different states reversibly. The transition conditions are in red font.

The reprogramming of the intelligence embedded in the aforementioned soft metamaterials typically requires manual reconnection, which can be cumbersome. To address this limitation and enable autonomous variation of the embedded intelligence, we have developed a soft metamaterial processor consisting of two SR latches, a 2–4 demultiplexer, and four ring oscillators, as depicted in **Figure** **8**a. The outputs of the two SR latches are input into the demultiplexer, where the 2 data lines are switched to 4 data lines. The resulting four outputs of the demultiplexer, denoted as *N*_1_, *N*_2_, *N*_3_, and *N*_4_, serve as the power source for the ring oscillators. These oscillators generate oscillatory pressures (*P*_1_, *P*_2_, *P*_3_, and *P*_4_), which are connected to four fingers, respectively. It should be noted that the processor and the fingers are only powered by a constant vacuum pressure.

As shown in Figure 8b,c and Movie S11 (Supporting Information), this processor can be transitioned between four different states reversibly, therefore enabling the execution of various fluidic programs without reconfiguring the connections between unit cells. In the initial state (Figure 8c(i)), the outputs of SR Latch‐1 and SR Latch‐2 are in the logic low state (*Q*_1_ = 0, *Q*_2_ = 0, ${\bar{Q}}_{1}$= 1, ${\bar{Q}}_{2}$= 1), resulting in the logic high state of port *N*_1_ ($N_{1}={\bar{Q}}_{1}{\bar{Q}}_{2}$). The vacuum pressure outputted from port *N*_1_ activates Ring Oscillator‐1, initiating periodic bending and extension of the first finger. Pressing unit cell “*S_2_*” transitions the processor into the second state (Figure 8c(ii)), where SR Latch‐1 outputs “0” and SR Latch‐2 outputs “1”, causing port *N*_2_ to become the logic high state ($N_{2}={\bar{Q}}_{1}Q_{2}$). Consequently, Ring Oscillator‐2 is activated, prompting periodic deformation of the second finger. Notably, the current state persists even after the removal of the pressing force, owing to the ability of the SR latches to retain their logic states until updated by new pressing forces.

Similarly, pressing unit cells “*S_1_*” and “*R_2_*” triggers the activation of Ring Oscillator‐3, inducing the periodic deformation of the third finger (Figure 8c(iii)). Subsequent application of pressing force on unit cell “*S_2_*” activates Ring Oscillator‐4, causing the fourth finger to deform periodically (Figure 8c(iv)). The state‐storing capability of the SR latches, the data distribution functionality of the demultiplexer, and the oscillatory behavior of the ring oscillators collectively allow the processor to autonomously execute multiple fluidic programs with only a constant vacuum pressure.

## 3 Conclusion and Discussion

We have demonstrated a reprogrammable, printable, and recyclable soft metamaterial composed of interconnected and periodically arranged logic unit cells. These unit cells possess the unique capability to perform compound logic operations coupling multiplication and negation, as well as scalable computation. Through the incorporation of the laws of physical Boolean algebra and a universal design route, these unit cells can be assembled into intelligent soft metamaterials with a wide range of functionalities, ranging from eight fundamental logic operations to advanced arithmetic operations. In order to showcase their ability to function as fluidic processors, we have successfully fabricated a soft latched demultiplexer, which is able to exert control over 2^n^−1 independent actuators using only n fluidic inputs. Additionally, we have engineered metamaterials‐based soft ring oscillators that enable autonomous regulation of both positive and negative pressures. The customizable morphing programming and autonomous reprogramming capabilities of the metamaterials are demonstrated by regulating a soft hand to exhibit different gestures.

The presented soft metamaterials showcase two distinctive features. i) The logic unit cell depends on the mechanical behavior coupled with a fluidic network to execute a logic operation that contains negation and multiplication. Both input and output signals are in the form of fluidic pressures, permitting compatible communications between different unit cells. This unique characteristic allows for the arrangement of multiple unit cells into intelligent soft metamaterials capable of directly executing complex logic functions and functioning as fluidic processors for soft robots. In addition, this coupling strategy can also be generalized to electrically‐driven soft actuators,^[^ ^49^, ^50^, ^51^ ^]^ allowing them to be equipped with embedded intelligence. ii) The soft metamaterials are able to exhibit reprogrammable functionalities through varying the connections between logic unit cells, which can satisfy on‐demand requirements in an unpredicted environment. Furthermore, the state‐storing capability of the SR latches, the data distribution functionality of the demultiplexer, and the oscillatory behavior of the ring oscillators collectively empower the processor to autonomously execute multiple fluidic programs without reconfiguring the connections between unit cells.

Compared with previous soft logic devices,^[^ ^52^, ^53^, ^54^ ^]^ our work has several advantages. i) The computation capacity of a single unit cell can be scaled by increasing the number of long tubes along the curved surface. This scalability empowers the unit cells to effectively regulate soft actuators driven by different pressure types and magnitudes. By combining the autonomous operation of the ring oscillator with the scalable computation capacity, a soft processor endowed with customizable morphing programming capability can be created. This advancement holds great promise for the execution of sophisticated and high‐level control operations. ii) The previous studies^[^ ^52^, ^53^, ^54^ ^]^ relied on the expertise of researchers to design fluidic circuits for soft robots, which can be challenging and time‐consuming for beginners. In contrast, our work establishes the laws of physical Boolean algebra and a universal design route, enabling the effortless construction of various complex and advanced fluidic circuits. iii) The logic unit cells can be directly printed with a commercial 3D printer, allowing for cost‐effective mass production. Furthermore, they can also be recycled without compromising their mechanical and logical properties, highlighting their potential contribution to a circular economy and reduction of material waste. iv) The presented logic unit cells are actuated by vacuum pressure, providing superior leak resistance and durability compared with those soft logic devices^[^ ^52^, ^53^, ^54^ ^]^ powered by pressurized air.

Although our current unit cells primarily focus on centimeter‐scale structures, recent advancements in microscale fabrication and actuation^[^ ^55^, ^56^, ^57^ ^]^ offer exciting prospects for extending this approach to microscale and nanoscale structures. Such expansions will enable the creation of soft metamaterials with novel properties and functions across a broad range of dimensions.

## 4 Experimental Section

### Analytical Model of the Logic Unit Cell

The logic unit cell is a fluidic component and was analyzed using the equivalent electric circuit illustrated in Figure S4a–c (Supporting Information). In this circuit, the fluidic pressure (Pa), mass flow rate (kg s^−1^), fluidic resistance (Pa·s kg^−1^), and fluidic capacity (kg Pa^−1^) exhibited a direct analogy to the voltage (V), current (A), resistance (Ω), and capacity (F) of an electric circuit, respectively. Tube‐1 and Tube‐2 denoted the elastomer tubes that connected ports *N*_1_ and *N*_2_. *R_1_*, *R_2_*, and *R_capi_* referred to the fluidic resistance of Tube‐1, Tube‐2, and the capillary tube, respectively. *C_cham_* and *C_3_* represented the fluidic capacity of the chamber of the unit cell and the airtight channel connecting with port *N*_3_, respectively. *P_cham_* and *P_3_* denoted the pressure of the chamber and port *N*_3_. *S*_1_ and *S*_2_ are mechanical switches that symbolize the states of ports *N*_1_ and *N*_3_, while *S*_K_ is a fluidic relay that denotes the state of the long tube between ports *N*_2_ and *N*_3_.

The kinking pressure of the unit cell is defined as *P_kink_* (Figure S3, Supporting Information). If the internal pressure of the chamber is higher than *P_kink_* (*P_cham_* > *P_kink_*), the relay *S*_K_ is in the “off” state (Figure S4a, Supporting Information) with port *N*_3_ subjected to the vacuum. Conversely, when *P_cham_* ≤ *P_kink_*, the relay *S*_K_ is switched to the “on” state (Figure S4b, Supporting Information) and port *N*_3_ is connected to the atmosphere.

The Reynolds number in the system was estimated to be in the range of 10–100, which is considerably lower than the critical Reynolds number (*Re* ≈2300) associated with the transition to turbulent flow. Consequently, the fluidic resistance *R_fluid_* was determined using the Darcy–Weisbach equation in the laminar flow regime:

(1) $$R_{fluid}=\frac{\Delta P}{\dot{m}}=\frac{128\mu L}{\pi \rho D^{4}}$$

where Δ*P* represents the pressure difference between the two ends of the fluidic tube, $\dot{m}$ is the mass flow rate of air,μ is the dynamic viscosity of air, ρ is the density of air, *D* is the internal diameter of the tube, and *L* is the length of the tube.

The fluidic capacity can be determined using the ideal gas equation of state:

(2) $$C_{fluid}=\frac{dm}{dP}=\frac{VM}{RT}$$

where *V* is the volume of the chamber or tube, *M* is the molar mass of air, *R* is the universal gas constant, and *T* is the temperature.

i. When the unit cell was switched from *N*_1_ *N*_2_ *N*_3_ = “011” (Figure S4a, Supporting Information) to *N*_1_ *N*_2_ *N*_3_ = “110” (Figure S4b, Supporting Information), the air flowed from the chamber to the vacuum source via Tube‐1. Once the pressure of the chamber, *P_cham_* reached the kinking pressure of the unit cell *P_kink_*, the air flowed from the atmosphere to the airtight channel connecting with port *N*_3_ through the capillary tube. Then the pressure of the chamber and port *N*_3_ can be described as:
(3) $$P_{S}=C_{cham}\frac{dP_{cham}}{dt}R_{1}+P_{cham}$$
(4) $$R_{capi}C_{3}\frac{dP_{3}}{dt}+P_{3}=0$$

With the condition *t* = 0*, P_cham_* = *P_atm_*; *t* = *t_kink_*, *P_3_* = *P_3H_*, the solutions to Equations (3) and (4) are:

(5) $$P_{cham}=\frac{P_{S}}{R_{1}C_{cham}}+e^{-\frac{1}{R_{1}C_{cham}}t}\left(P_{atm}-\frac{P_{S}}{R_{1}C_{cham}}\right)$$

(6) $$P_{3}=\left\{\begin{array}{cc} P_{3H} & t<t_{kink}\\ P_{3H}e^{-\frac{1}{R_{capi}C_{3}}\left(t-t_{kink}\right)} & t\geq t_{kink} \end{array}\right.$$

(7) $$P_{3H}=\frac{R_{capi}}{R_{2}+R_{capi}}P_{S}$$

(8) $$t_{kink}=-R_{1}C_{cham}\ln\frac{R_{1}C_{cham}P_{kink}-P_{S}}{R_{1}C_{cham}P_{atm}-P_{S}}$$

where *P_3H_* denotes the pressure of port *N*_3_ in the logic “High” state.

ii. During the transition from *N*_1_ *N*_2_ *N*_3_ = “110” (Figure S4b, Supporting Information) to *N*_1_ *N*_2_ *N*_3_ = “011” (Figure S4a, Supporting Information), air entered the chamber via Tube‐1. Once the internal pressure of the chamber exceeded *P_kink_*, the long tube opened, resulting in the evacuation of the air from port *N*_3_. The pressure of the chamber and port *N*_3_ can be described as:
(9) $$R_{1}C_{cham}\frac{dP_{cham}}{dt}+P_{cham}=0$$
(10) $$\left(\frac{P_{3}}{R_{capi}}+C_{3}\frac{dP_{3}}{dt}\right)R_{2}+P_{3}=P_{S}$$

With the condition *t* = 0, *P_cham_* = *P_S_*; *t* = *t_open_*, *P_3_* = *P_atm_*, the solutions to Equations (9) and (10) are:

(11) $$P_{cham}=P_{S}e^{-\frac{1}{R_{1}C_{cham}}t}$$

(12) $$P_{3}=\left\{\begin{array}{c} P_{atm}t<t_{open}\\ X_{2}+e^{-X_{1}\left(t-t_{open}\right)}\left(P_{atm}-X_{2}\right)t\geq t_{open} \end{array}\right.$$

(13) $$X_{1}=\frac{R_{2}+R_{capi}}{R_{2}R_{capi}C_{3}},\;X_{2}=\frac{P_{S}}{R_{2}C_{3}}$$

(14) $$t_{open}=-R_{1}C_{cham}\ln\frac{P_{open}}{P_{S}}$$

where *P_open_* is the threshold pressure that the long tube is switched from a kinked state to an open state.

The response time required to complete the transition between *N*_1_ *N*_2_ *N*_3_ = “011” and *N*_1_ *N*_2_ *N*_3_ = “110” of the unit cell can be calculated by Equation (6) and (12), respectively.

(15) $$t_{011\to 110}=-R_{capi}C_{3}\ln\frac{P_{3A}}{P_{3H}}+t_{kink}$$

(16) $$P_{3A}=\left(P_{3H}-P_{atm}\right)\times 10\%+P_{atm}$$

(17) $$t_{110\to 011}=-\frac{1}{X_{1}}\ln\frac{P_{3B}-X_{2}}{P_{atm}-X_{2}}+t_{open}$$

(18) $$P_{3B}=\left(P_{3H}-P_{atm}\right)\times 90\%+P_{atm}$$

The state that *P_3_* ≥ *P_3A_* is defined as the logic “Low” state, while the state that *P_3_* ≤ *P_3B_* is defined as the logic “High” state.

iii. When the unit cell was switched from *N*_1_ *N*_2_ *N*_3_ = “000” (Figure S4c, Supporting Information) to *N*_1_ *N*_2_ *N*_3_ = “011” (Figure S4a, Supporting Information), the air flowed from the atmosphere to port *N*_2_ via the capillary tube. Then the pressure of port *N*_3_ can be expressed as:
(19) $$\left(\frac{P_{3}}{R_{capi}}+C_{3}\frac{dP_{3}}{dt}\right)R_{2}+P_{3}=P_{S}$$

With the condition *t* = 0, *P_3_* = *P_atm_*, the solution to Equation (19) is:

(20) $$P_{3}=X_{2}+e^{-X_{1}t}\left(P_{atm}-X_{2}\right)$$

(21) $$X_{1}=\frac{R_{2}+R_{capi}}{R_{2}R_{capi}C_{3}},\;X_{2}=\frac{P_{S}}{R_{2}C_{3}}$$

iv. When the unit cell was switched from *N*_1_ *N*_2_ *N*_3_ = “011” (Figure S4a, Supporting Information) to *N*_1_ *N*_2_ *N*_3_ = “000” (Figure S4c, Supporting Information), air flowed from the atmosphere to port *N*_3_ mainly through Tube‐2. Then the pressure of port *N*_3_ can be expressed as:
(22) $$R_{2}C_{3}\frac{dP_{3}}{dt}+P_{3}=0$$

With the condition *t* = 0*, P_3_* = *P_3H_*, the solution to Equation (22) is:

(23) $$P_{3}=P_{3H}e^{-\frac{1}{R_{2}C_{3}}t}$$

(24) $$P_{3H}=\frac{R_{capi}}{R_{2}+R_{capi}}P_{S}$$

The response time required to complete the transition between *N*_1_ *N*_2_ *N*_3_ = “011” and *N*_1_ *N*_2_ *N*_3_ = “000” of the unit cell can be calculated by Equation (20) and (23), respectively.

(25) $$t_{000\to 011}=-\frac{1}{X_{1}}\ln\frac{P_{3B}-X_{2}}{P_{atm}-X_{2}}$$

(26) $$t_{011\to 000}=-R_{2}C_{3}\ln\frac{P_{3A}}{P_{3H}}$$

In this study, the elastomer tube with an internal diameter of 2.0 mm and an external diameter of 3.0 mm was selected to establish the channel between ports *N*_2_ and *N*_3_, while the elastomer tube for port *N*_1_ had an internal diameter of 1.5 mm and an external diameter of 3.0 mm. The tubes with this dimension allowed the unit cell to exhibit a relatively fast response, resulting in a considerably small response time for *t_110→011_*, *t_000→011_*, and *t_011→000_* (< 0.5 s). In contrast, capillary tubes exhibited high fluidic resistance, which significantly affected the response characterization of the unit cell. Therefore, this analytical model is employed to calculate *t_011→110_* of the unit cell. The results are presented in Figure S4d,e (Supporting Information), demonstrating excellent agreement between the calculated response time and experimental values. This agreement illustrated the potential of the analytical model in analyzing complex soft fluidic processors constructed with the presented unit cells.

### Fabrication of the Logic Unit Cell

The logic unit cells were fabricated in a 3D printer (Hori H2, Beijing Huitianwei Technology) with TPU filament (55A, Yitailong High‐tech Materials). Then elastomer tubes (2 × 3 mm, Shanghai Daoguan Xiangsu Wujin Co. Ltd.) were plugged into the unit cell. The logic unit cells were recycled in a plastic extrusion machine (B2, Wellzoom).

### Fabrication of the Soft Hand and Bending Actuators

The soft hand and bending actuators were fabricated using the elastomer casting technique. All the molds for elastomer casting were made of polylactic acid (PLA) and manufactured with a 3D printer (Trianglelab, Dforce 300). The fabrication process involved four steps: i) Two components of elastomers (E620, Shenzhen Hongyejie Technology Co., Ltd.) and pigment were mixed using a glass rod. The mixture was then degassed in a vacuum container to remove the air in it. ii) The prepared elastomer mixtures were poured into the PLA molds. Subsequently, the molds were placed in an oven (DZF‐6090AB, Lichen) and heated at 65 °C for 30 min to facilitate curing. iii) The cured elastomers were removed from the molds. iv) The elastomer components were stuck together using silicone adhesive (Sil‐Poxy, Smooth‐on).

### Control and Measuring System

To regulate and measure the pressure traces of both the logic unit cells and soft metamaterials, a custom‐built control and measuring system was constructed. As illustrated in Figure S8 (Supporting Information), the logic state of the two input ports of the logic unit cell was controlled by solenoid valves (KVE32PL24FF, Kamoer). The electrical relays received control signals from an Arduino board (MEGA2560 R3) and converted them into electrical signals that could actuate the solenoid valves directly. The input pressures for the unit cell were generated by a vacuum pump (V‐I240SV pump, VALUE) and regulated with a pressure regulator (ITV 2090, SMC). The pressure magnitudes of the input and output ports were measured with pressure sensors (XGZP6847A, CFSensor). The resulting output signals from these sensors were transmitted to a computer via a home‐built data acquisition system. The positive pressures were produced by an air compressor (DET750, DAERTUO) and regulated by a pressure regulator (ITV 1030, SMC). The bending angle of the logic unit cell was measured with an inclination sensor (MMA8451).

## Conflict of Interest

The authors declare no conflict of interest.

## Supporting information

Supporting Information

Supplemental Movie 1

Supplemental Movie 2

Supplemental Movie 3

Supplemental Movie 4

Supplemental Movie 5

Supplemental Movie 6

Supplemental Movie 7

Supplemental Movie 8

Supplemental Movie 9

Supplemental Movie 10

Supplemental Movie 11

## Data Availability

The data that support the findings of this study are available from the corresponding author upon reasonable request.

## References

[1] K. Bertoldi , V. Vitelli , J. Christensen , M. Van Hecke , Nat. Rev. Mater. 2017, 2, 17066.

[2] R. Khajehtourian , D. M. Kochmann , Front. Robot. AI 2021, 8, 673478. 34012982 10.3389/frobt.2021.673478 PMC8126663

[3] X. Yu , J. Zhou , H. Liang , Z. Jiang , L. Wu , Prog. Mater. Sci. 2018, 94, 114.

[4] Y. Li , Q. Zhang , Y. Hong , J. Yin , Adv. Funct. Mater. 2021, 31, 2105641.

[5] Y. Shi , Q. Song , I. Toftul , T. Zhu , Y. Yu , W. Zhu , D. P. Tsai , Y. Kivshar , A. Q. Liu , Appl. Phys. Rev. 2022, 9, 031303.

[6] J. Li , K. F. Macdonald , N. I. Zheludev , Nano Lett. 2022, 22, 4301. 35609218 10.1021/acs.nanolett.1c04900 PMC9185736

[7] N. Gao , Z. Zhang , J. Deng , X. Guo , B. Cheng , H. Hou , Adv. Mater. Technol. 2022, 7, 2100698.

[8] G. Ji , J. Huber , Appl. Mater. Today 2022, 26, 101260.

[9] Z. Tao , X. Ren , A. G. Zhao , L. Sun , Y. Zhang , W. Jiang , D. Han , X. Y. Zhang , Y. M. Xie , Int J Mech Sci 2022, 226, 107414.

[10] R. Ju , G. Xu , L. Xu , M. Qi , D. Wang , P.‐C. Cao , R. Xi , Y. Shou , H. Chen , C.‐W. Qiu , Y. Li , Adv. Mater. 2023, 35, 2209123. 10.1002/adma.202209123 36621882

[11] H. Yu , H. Wang , X. Guo , B. Liang , X. Wang , H. Zhou , X. Zhang , M. Chen , H. Lei , Compos. Struct. 2022, 300, 116131.

[12] Z. Zheng , Y. Luo , H. Yang , Z. Yi , J. Zhang , Q. Song , W. Yang , C. Liu , X. Wu , P. Wu , Phys. Chem. Chem. Phys. 2022, 24, 8846. 35356962 10.1039/d2cp01070d

[13] X. Fang , J. Wen , L. Cheng , D. Yu , H. Zhang , P. Gumbsch , Nat. Mater. 2022, 21, 869. 35681063 10.1038/s41563-022-01269-3 PMC9345786

[14] M. J. Mirzaali , S. Janbaz , M. Strano , L. Vergani , A. A. Zadpoor , Sci. Rep. 2018, 8, 965. 29343772 10.1038/s41598-018-19381-3 PMC5772660

[15] T. Chen , M. Pauly , P. M. Reis , Nature 2021, 589, 386. 33473228 10.1038/s41586-020-03123-5

[16] J. T. B. Overvelde , T. A. De Jong , Y. Shevchenko , S. A. Becerra , G. M. Whitesides , J. C. Weaver , C. Hoberman , K. Bertoldi , Nat. Commun. 2016, 7, 10929. 26965475 10.1038/ncomms10929 PMC4793042

[17] H. Cui , D. Yao , R. Hensleigh , H. Lu , A. Calderon , Z. Xu , S. Davaria , Z. Wang , P. Mercier , P. Tarazaga , X. (.R.). Zheng , Science 2022, 376, 1287. 35709267 10.1126/science.abn0090

[18] Q. Pan , S. Chen , F. Chen , X. Zhu , Sci. China Technol. Sci. 2020, 63, 2518.

[19] A. Rafsanjani , K. Bertoldi , A. R. Studart , Sci. Robot. 2019, 4, eaav7874. 33137714 10.1126/scirobotics.aav7874

[20] S. Jiang , X. Liu , J. Liu , D. Ye , Y. Duan , K. Li , Z. Yin , Y. Huang , Adv. Mater. 2022, 34, 2200070. 10.1002/adma.202200070 35325478

[21] H. Pei , J. Jing , Y. Chen , J. Guo , N. Chen , Nano Energy 2023, 109, 108303.

[22] X. Huang , W. Guo , S. Liu , Y. Li , Y. Qiu , H. Fang , G. Yang , K. Zhu , Z. Yin , Z. Li , H. Wu , Adv. Funct. Mater. 2022, 32, 2109109.

[23] R. Zhang , Q. Chen , K. Liu , Z. Chen , K. Li , X. Zhang , J. Xu , E. Pickwell‐Macpherson , IEEE Trans. Terahertz Sci. Technol. 2019, 9, 209.

[24] Y. Cao , K. Chen , C. Ruan , X. Zhang , Sens. Actuator A Phys. 2021, 331, 112869.

[25] X. Xu , D. Zheng , Y.‐S. Lin , J. Colloid Interface Sci. 2023, 642, 462. 37023517 10.1016/j.jcis.2023.03.190

[26] Y. Bao , G. Hong , Y. Chen , J. Chen , H. Chen , W.‐L. Song , D. Fang , ACS Appl. Mater. Interfaces 2020, 12, 780. 31849209 10.1021/acsami.9b18232

[27] D. G. Mackanic , M. Kao , Z. Bao , Adv. Energy Mater. 2020, 10, 2001424.

[28] Z. Jiao , C. Zhang , J. Ruan , W. Tang , Y. Lin , P. Zhu , J. Wang , W. Wang , H. Yang , J. Zou , Cell Rep Phys Sci 2021, 2, 100407.

[29] J. Zhang , L. Liu , Y. Chen , M. Zhu , L. Tang , C. Tang , J. Shintake , J. Zhao , J. He , X. Ren , P. Li , Q. Huang , H. Zhao , J. Lu , D. Li , Cell Rep Phys Sci 2021, 2, 100600.

[30] Z. Jiao , C. Zhang , W. Wang , M. Pan , H. Yang , J. Zou , Adv. Sci. 2019, 6, 1901371. 10.1002/advs.201901371 PMC6839643 31728286

[31] Z. Lin , Q. Shao , X.‐J. Liu , H. Zhao , Adv. Intell. Syst. 2022, 4, 2200126.

[32] Z. Jiao , P. Zhu , Z. Hu , Y. Shi , W. Tang , H. Yang , J. Zou , Adv. Mater. Technol. 2023, 8, 2300476.

[33] Z. Jiao , C. Ji , J. Zou , H. Yang , M. Pan , Adv. Mater. Technol. 2019, 4, 1800429.

[34] M. Wehner , R. L. Truby , D. J. Fitzgerald , B. Mosadegh , G. M. Whitesides , J. A. Lewis , R. J. Wood , Nature 2016, 536, 451. 27558065 10.1038/nature19100

[35] A. Rafsanjani , Y. Zhang , B. Liu , S. M. Rubinstein , K. Bertoldi , Sci Robot 2018, 3, eaar7555. 33141681 10.1126/scirobotics.aar7555

[36] A. Rafsanjani , L. Jin , B. Deng , K. Bertoldi , Proc. Natl. Acad. Sci. USA 2019, 116, 8200. 30962388 10.1073/pnas.1817763116 PMC6486746

[37] A. G. Mark , S. Palagi , T. Qiu , P. Fischer , IEEE Int. Conf. on Robotics and Automation, Stockholm, Sweden May, 2016.

[38] P. Bhovad , J. Kaufmann , S. Li , Extreme Mech Lett 2019, 32, 100552.

[39] D. Melancon , A. E. Forte , L. M. Kamp , B. Gorissen , K. Bertoldi , Adv. Funct. Mater. 2022, 32, 2201891.

[40] T. Mei , Z. Meng , K. Zhao , C. Q. Chen , Nat. Commun. 2021, 12, 7234. 34903754 10.1038/s41467-021-27608-7 PMC8668933

[41] C. El Helou , P. R. Buskohl , C. E. Tabor , R. L. Harne , Nat. Commun. 2021, 12, 1633. 33712597 10.1038/s41467-021-21920-y PMC7954845

[42] H. Zhang , J. Wu , D. Fang , Y. Zhang , Sci. Adv. 2021, 7, eabf1966. 33627434 10.1126/sciadv.abf1966 PMC7904272

[43] C. El Helou , B. Grossmann , C. E. Tabor , P. R. Buskohl , R. L. Harne , Nature 2022, 608, 699. 36002486 10.1038/s41586-022-05004-5

[44] Y. Jiang , L. M. Korpas , J. R. Raney , Nat. Commun. 2019, 10, 128. 30631058 10.1038/s41467-018-08055-3 PMC6328580

[45] Z. Ren , L. Ji , R. Tao , M. Chen , Z. Wan , Z. Zhao , D. Fang , Extreme Mech. Lett. 2021, 42, 101077.

[46] U. Waheed , C. W. Myant , S. N. Dobson , Extreme Mech Lett 2020, 40, 100865.

[47] R. Ma , L. Wu , D. Pasini , Adv. Funct. Mater. 2023, 33, 2213371.

[48] D. Ricketts , J. A. McNeill , The Designer's Guide to Jitter in Ring Oscillators, Springer, Berlin, Germany, 2009.

[49] Z. Mao , T. Iizuka , S. Maeda , Sens. Actuator A Phys. 2021, 332, 113168.

[50] C. Tang , B. Du , S. Jiang , Q. Shao , X. Dong , X.‐J. Liu , H. Zhao , Sci Robot 2022, 7, eabm8597. 35613300 10.1126/scirobotics.abm8597

[51] Z. Mao , Y. Peng , C. Hu , R. Ding , Y. Yamada , S. Maeda , Biomimetic Intell. Robot. 2023, 3, 100114.

[52] D. J. Preston , H. J. Jiang , V. Sanchez , P. Rothemund , J. Rawson , M. P. Nemitz , W.‐K. Lee , Z. Suo , C. J. Walsh , G. M. Whitesides , Sci Robot 2019, 4, eaaw5496. 33137768 10.1126/scirobotics.aaw5496

[53] D. Drotman , S. Jadhav , D. Sharp , C. Chan , M. T. Tolley , Sci Robot 2021, 6, eaay2627. 34043527 10.1126/scirobotics.aay2627

[54] D. J. Preston , P. Rothemund , H. J. Jiang , M. P. Nemitz , J. Rawson , Z. Suo , G. M. Whitesides , Proc. Natl. Acad. Sci. USA 2019, 116, 7750. 30923120 10.1073/pnas.1820672116 PMC6475414

[55] E. Leroy , R. Hinchet , H. Shea , Adv. Mater. 2020, 32, 2002564. 10.1002/adma.202002564 32700326

[56] H. Zhao , A. M. Hussain , A. Israr , D. M. Vogt , M. Duduta , D. R. Clarke , R. J. Wood , Soft Robot 2020, 7, 451. 31923364 10.1089/soro.2019.0113

[57] L. Hines , K. Petersen , G. Z. Lum , M. Sitti , Adv. Mater. 2016, 29, 1603483. 10.1002/adma.201603483 28032926

