# Supplementary material for: Reprogrammable Metamaterial Processors for Soft Machines
Source: Adv Sci (Weinh). 2023 Dec 31;11(11):2305501. doi: 10.1002/advs.202305501 (PMC10953550; doi:10.1002/advs.202305501)
Supplement: Supplementary file 1 — Supporting Information [file ADVS-11-2305501-s010.pdf]

ADVANCED SCIENCE
Open Access

Supporting Information

for *Adv. Sci.*, DOI 10.1002/advs.202305501

Reprogrammable Metamaterial Processors for Soft Machines

*Zhongdong Jiao, Zhenhan Hu, Zeyu Dong, Wei Tang, Huayong Yang and Jun Zou**

Supporting Information

Reprogrammable metamaterial processors for soft machines

Zhongdong Jiao[1], Zhenhan Hu[1], Zeyu Dong[1], Wei Tang[1], Huayong Yang[1], Jun Zou[1]*

[1]State Key Laboratory of Fluid Power and Mechatronic Systems, Zhejiang University, Hangzhou 310058, China.

Email of Corresponding Author: junzou@zju.edu.cn

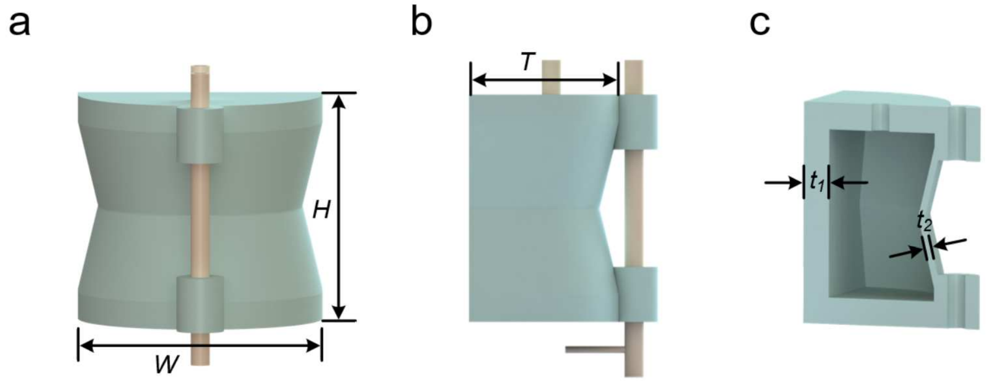

Figure S1. The dimensions and internal structure of the logic unit cell. a The front view. H = 33 mm, W = 36 mm. **b** The side view. T = 22 mm. **c** The cross-section view. t_1 = 4 mm, t_2 = 1 mm.

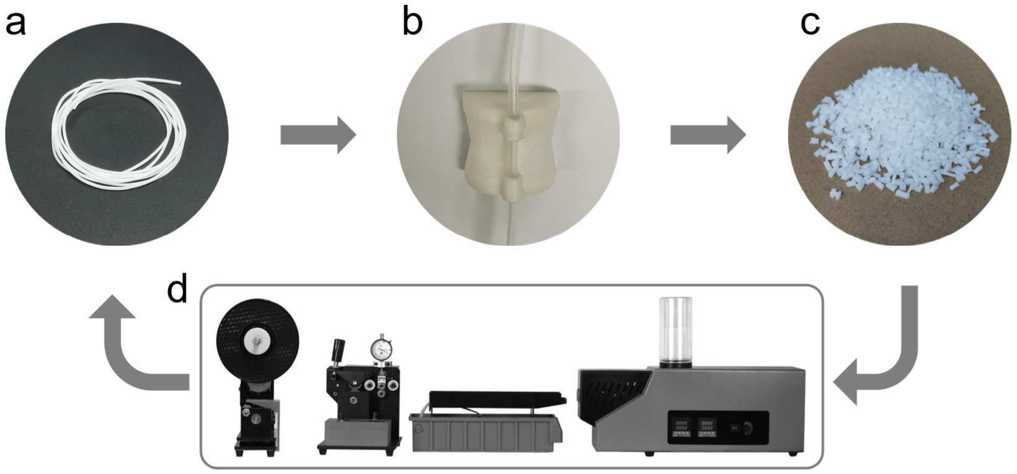

Figure S2. The recycling process of the logic unit cell. a The filament wires produced by the plastic extrusion machine. **b** The logic unit cell printed with the filament wires by a 3D printer. **c** The logic unit cell is cut into small pieces. **d** The small pieces are fed into a plastic extrusion machine. The machine melts and converts the plastic fragments into continuous filament wires.

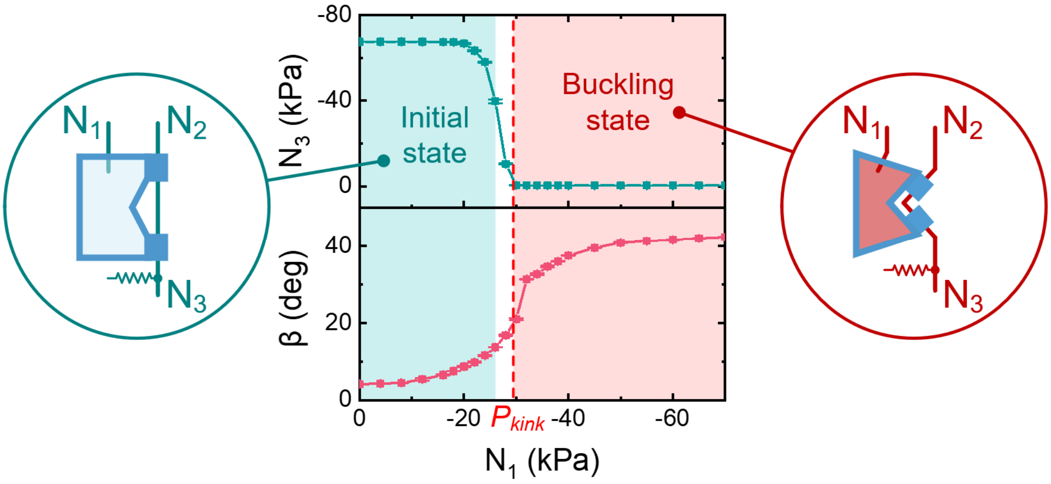

Figure S3. The pressure of port N_3 and bending angle of the unit cell as a function of the pressure of port N_1.

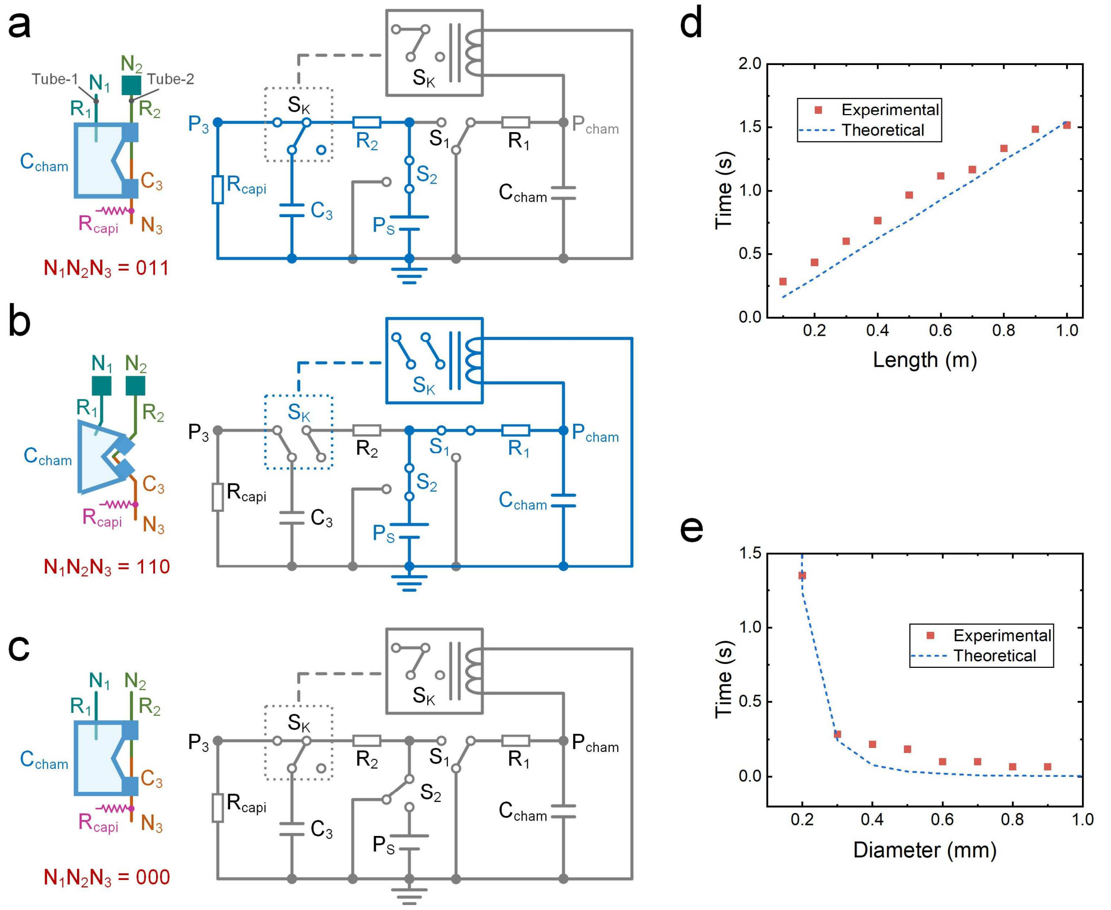

Figure S4. The analytical model of the logic unit cell. a-c The logic unit cell can be analyzed using an equivalent fluidic circuit. (**a**) The input ports N_1 and N_2 are connected to atmospheric pressure and vacuum pressure, respectively. (**b**) The input ports N_1 and N_2 are connected to vacuum pressure. (**c**) The input ports N_1 and N_2 are connected to atmospheric pressure. **d** The logic response time of the unit cell as a function of the length of capillary tube. **e** The logic response time of the unit cell as a function of the internal diameter of capillary tube.

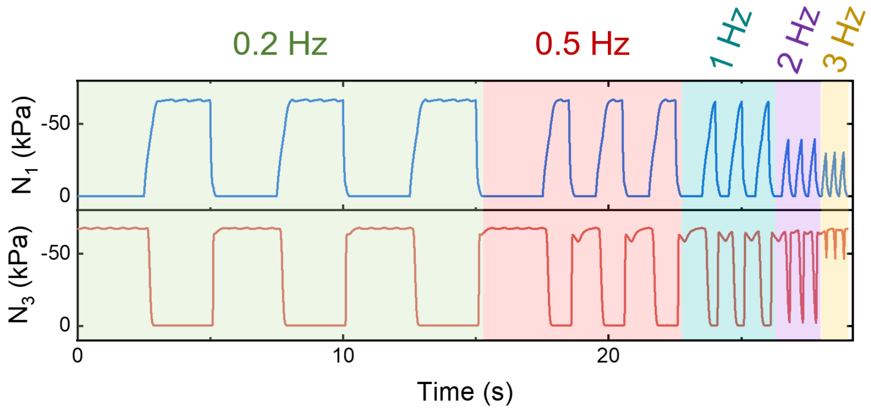

Figure S5. The logic unit cell is actuated at a frequency ranging from 0.2 Hz to 3 Hz.

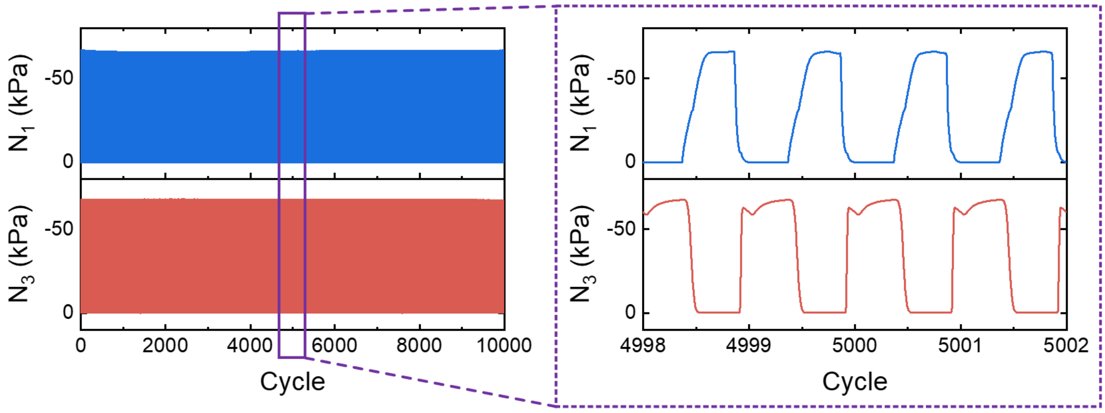

Figure S6. The logic fatigue test of the unit cell. A unit cell is operated 10,000 times under a frequency of 0.5 Hz. The actuation pressure is -70 kPa.

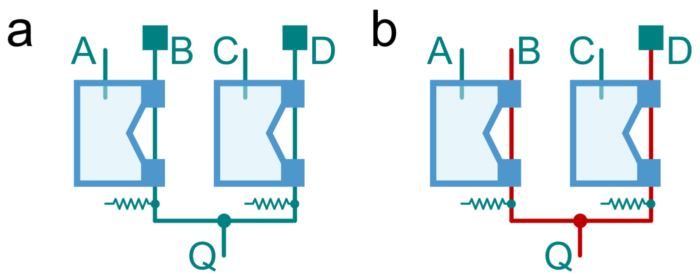

Figure S7. The logic expression $Q = \overline{A}B + \overline{C}D$ is physicalized in parallel form. a Input ports B and D are connected to vacuum pressure, the generated results are correct. **b** Input Ports A, B, and C are connected to atmospheric pressure, and input port D is connected to vacuum pressure, the output port Q is supposed to be in a logic “1” state. However, the parallel connection interconnects ports B and D, causing the vacuum source to be connected to the atmosphere. Consequently, port Q becomes logic “0” state, which is a false output.

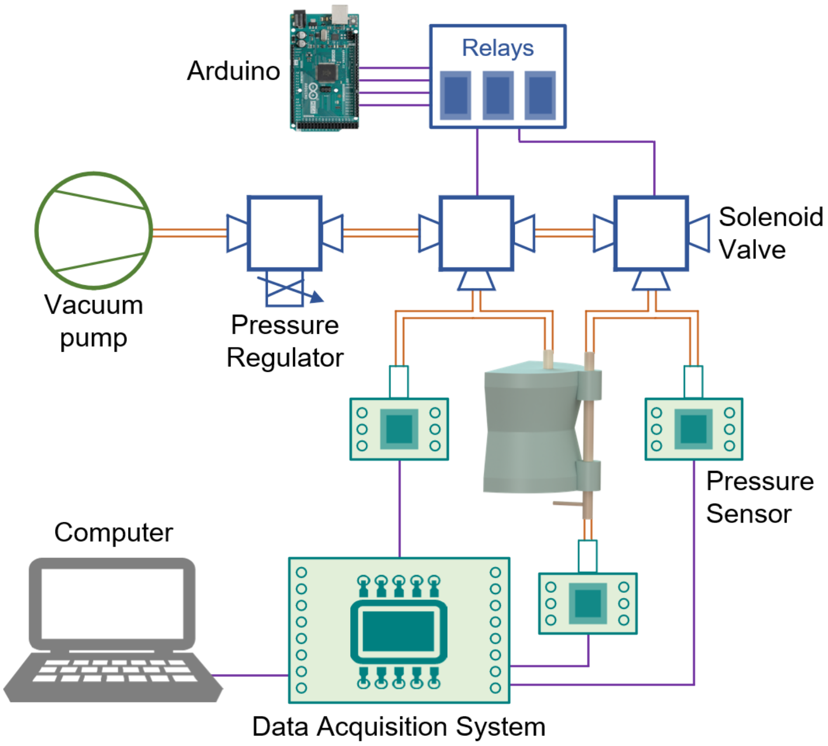

Figure S8. The control and measuring system of the logic unit cells.

Supplemental Movie Captions

Movie S1. The compound logic operation of the unit cell. The vacuum pressure and atmospheric pressure are defined as logic state "1" and "0", respectively.

Movie S2. The recycling process of the logic unit cell. The logic unit cell is cut into small pieces and fed into a plastic extrusion machine, where the small pieces are converted into continuous filament wires.

Movie S3. The scalable computation capacity of the unit cell. A logic unit cell equipped with four long tubes is able to execute four logic operations simultaneously.

Movie S4. The fundamental logic gates enabled by the soft logic unit cells. The logic unit cells are utilized to construct NOT, Buffer, OR, AND, NOR, NAND, XOR, and XNOR gates according to the laws of physical Boolean algebra.

Movie S5. The soft metamaterial-based full adder. Eight possible configurations of the full adder are validated in the physical metamaterial.

Movie S6. The soft metamaterial-based full subtractor. Eight possible configurations of the full subtractor are validated in the physical metamaterial.

Movie S7. The soft metamaterial-based demultiplexer. The demultiplexer acts as the fluidic processor for a soft machine with eight bending actuators.

Movie S8. The soft metamaterial-based latched demultiplexer. The latched demultiplexer is able to set seven soft actuators to any desired combination of actuated or unactuated states. In the first combination, the 1st, 2nd, and 3rd actuators are in actuated states. In the second combination, the 4th, 5th, 6th, and 7th actuators are in actuated states. In the third combination, the 1st, 3rd, 5th, and 7th actuators are in actuated states.

Movie S9. Autonomous soft controller. A soft five-stage ring oscillator functions as the autonomous controller capable of regulating a vacuum-powered soft hand and a positive pressure-driven soft hand.

Movie S10. Customizable morphing programming of the metamaterial processors. The soft hand exhibits six different gestures through the customizable morphing programming of the metamaterial processors.

Movie S11. Autonomously reprogrammable soft metamaterial processor. The soft hand exhibits six different gestures through the customizable morphing programming of the metamaterial processors. The processor can be reversibly transitioned between four different states without reconfiguring the connections between unit cells.
